# Using a flashlight-contingent window paradigm to investigate visual search and object memory in virtual reality and on computer screens

**DOI:** 10.1038/s41598-024-58941-8

**Published:** 2024-04-13

**Authors:** Julia Beitner, Jason Helbing, Erwan Joël David, Melissa Lê-Hoa Võ

**Affiliations:** 1 Department of Psychology, Goethe University Frankfurt, Frankfurt am Main, Germany; 2 LIUM, Le Mans Université, Le Mans, France

**Keywords:** Visual search, Incidental memory, Virtual reality, Ecological validity, Human behaviour, Long-term memory, Spatial memory, Attention, Perception

## Abstract

A popular technique to modulate visual input during search is to use gaze-contingent windows. However, these are often rather discomforting, providing the impression of visual impairment. To counteract this, we asked participants in this study to search through illuminated as well as dark three-dimensional scenes using a more naturalistic flashlight with which they could illuminate the rooms. In a surprise incidental memory task, we tested the identities and locations of objects encountered during search. Importantly, we tested this study design in both immersive virtual reality (VR; Experiment 1) and on a desktop-computer screen (Experiment 2). As hypothesized, searching with a flashlight increased search difficulty and memory usage during search. We found a memory benefit for identities of distractors in the flashlight condition in VR but not in the computer screen experiment. Surprisingly, location memory was comparable across search conditions despite the enormous difference in visual input. Subtle differences across experiments only appeared in VR after accounting for previous recognition performance, hinting at a benefit of flashlight search in VR. Our findings highlight that removing visual information does not necessarily impair location memory, and that screen experiments using virtual environments can elicit the same major effects as VR setups.

## Introduction

Visual search is the cognitive process of scanning a visual environment to detect a specific object (i.e., target) among other objects (i.e., distractors). This fundamental process plays a pivotal role in everyday human behavior and its importance is recognized with a wealth of research studying its mechanisms^1–3^. Beyond its intrinsic interest as a cognitive process, the study of visual search unveils additional layers of complexity warranting investigation. For example, visual search leads to incidental memory formation as a valuable byproduct, that is, long lasting memories which were not built on purpose, but can be recalled and used later on^4–6^. Exploring an environment through searching facilitates familiarization and enables proactive engagement within its context. For example, looking for a fork in a drawer of your friend’s kitchen might have led you to stumble over a corkscrew in the same drawer, which you incidentally encode and which might become useful later in the evening. Thus, two main features become behaviorally important here: object identity and object location. In other words, “what” (i.e., a corkscrew) can be found “where” (i.e., in the drawer). Incidental object identity and object location memory have both been studied in two-dimensional computer screen experiments^6–9^ as well as in three-dimensional immersive virtual^10,11^ and real-world environments^12,13^. Eye-tracking has shown that objects that were fixated longer were also remembered better later on^6,8,10–12^, indicating the importance of visual encoding. Moreover, research using varying tasks has established that not only visual encoding is of importance but that action-oriented behavior such as searching boosts memory to a greater degree than free viewing and even explicit memorization^9,11,12,14,15^.

One way to study the mechanisms of visual search in its complexity is to disrupt the cognitive process and observe how it adapts to the new circumstances. This can be achieved, for example, by limiting visual input and thereby increasing search difficulty, resulting in higher error rates and slower response times^3,16,17^. Limiting visual input can be implemented by using mouse- or gaze-contingent windows. Contingent windows only reveal a certain amount of information (most often a circle of varying degrees of visual angle) and follow the mouse cursor or the participant’s gaze as measured with eye-tracking. This type of research provides valuable insights into visual search mechanisms^18^, the different roles central and peripheral processing play^19,20^, and how well this translates to patients with vision loss (e.g., scotomas and tunnel vision)^21^.

In order to study these mechanisms during natural, everyday behavior in healthy humans, we also need to provide those situations in which participants can exhibit said behavior^22,23^. One possible solution to better capture daily life behaviors and to increase the ecological validity of experimental studies is to conduct experiments in virtual reality (VR). Researchers often face the so-called “real-world or the lab”-dilemma^24^, where ecological validity and high experimental control are perceived as opposing ends of a spectrum. VR, however, offers a promising solution to this challenge. It enables more unconstrained and naturalistic task settings while simultaneously maintaining a high level of experimental control^25–28^. The power of this approach has already been demonstrated in recent studies showing that effects found in conventional laboratory setups were weaker or even absent when tested in more realistic settings, which often engage behavior that is multi-modal, immersive, and self-referential^29–36^.

However, shifting away from computer screen studies to VR or even real-life environments will in many circumstances not be feasible or desired, as more realistic settings are often more demanding in terms of technical requirements and resources. Beyond that, computer screen studies may suffice and often capture and measure the same processes underlying natural human behavior. For example, a recent study on visual search has shown that search performance of T’s among L’s, a classic visual search paradigm^3^, predicts visual search performance in naturalistic scenes in VR^37^, thereby providing evidence that artificial search displays on computer screens do elicit behavior that is also present in more lifelike setups. Such studies are fundamental to ensure a balanced fit between behavior which is to be explained and behavior elicited in the laboratory, and in consequence whether behavior observed in on-screen studies generalizes to more realistic setups.

In our case, we developed a flashlight paradigm in VR^18^, in which a virtual flashlight is attached to a handheld controller and participants need to search for objects in a dark scene. Using a flashlight better resembles healthy participants’ personal experience than gaze-contingent windows. Humans, when navigating through dark environments in real life, often rely on handheld light sources (e.g., torch, flashlight, mobile phone), making this setup inherently more familiar and ecologically valid. This familiarity potentially reduces the cognitive load associated with adapting to an unusual visual experience and minimizes the risk of observing laboratory artifacts, allowing for a more accurate assessment of natural search behaviors and memory processes.

Here, we wanted to put the flashlight paradigm to the test and investigate (1) to what extent searching with a flashlight impacts search performance in terms of error rates and search times, and (2) how it influences incidental memory formation of object identities and locations. We hypothesized that using a flashlight in dark scenes would lead to decreased accuracy rates and longer search times compared to illuminated scenes. With regards to object memory, we hypothesized that both identity and location memory performance would be better for targets than for distractors, replicating previous findings^6,11,14^. However, the influence of increased search difficulty induced by the flashlight on incidental memory is unclear. One could argue that the increased search difficulty should lead to worse object memory or to the contrary: The forced processing of the central field of view might even increase object memory. With regards to object location memory, it might be that the increased search difficulty prevents the formation of spatial representation of the scene layout, resulting in an overall decreased memory of object locations.

**Figure 1:**
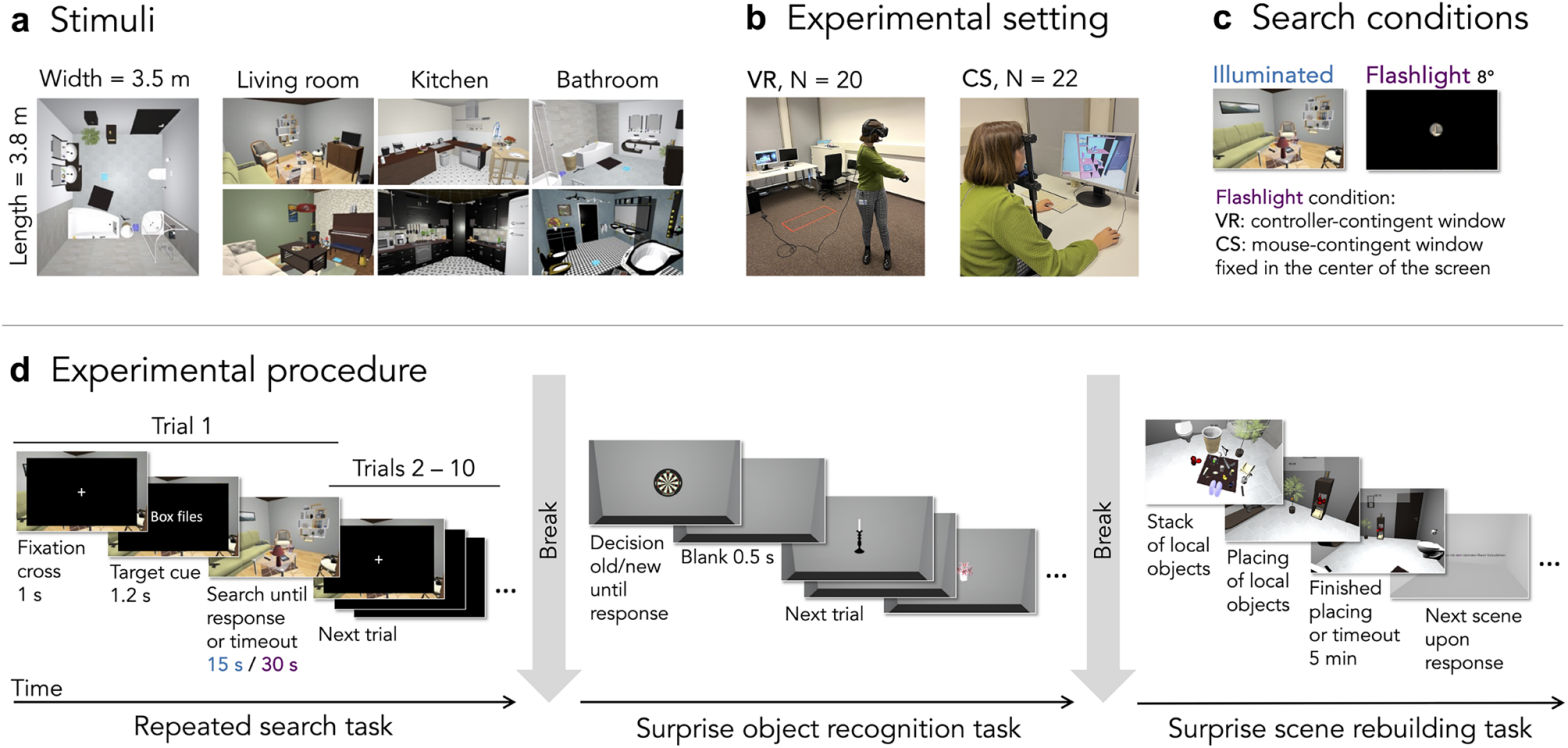
Overview of the experimental design. (**a**) Six 3D indoor scenes with eight global and 20 local objects each were used as stimuli. The image on the left depicts a bird’s-eye view of one of the bathrooms and the six other images depict a sample view of each scene. Blue squares indicate the starting position of the participants and were not visible during search. (**b**) Re-staged photographs of the two experimental settings VR and CS. The room light was dimmed during the actual experiments. (**c**) Search conditions of the repeated search task. The flashlight window in the figure approximates the actual diameter of 8$^{\circ }$. (**d**) Paradigm of the entire experimental procedure. Participants started with the repeated search task. Note that fixation cross and target cue are enlarged for enhanced visibility. After completion of the search task, participants could take a break and were informed and instructed about the first surprise task. Again, after completion, participants could take another break and were informed and instructed about the surprise scene rebuilding task. *CS* = computer screen, *VR* = virtual reality.

To test the flashlight paradigm, we conducted two experiments, in each of which participants first had to search successively for 10 out of 20 objects in total (i.e., 10 targets and 10 distractors, respectively) through six indoor scenes. Targets and distractors were small, local objects^38^, such as a remote control, a tennis racket or a rubber duck. Objects were assigned as target or distractor randomly for each participant. Scenes were either fully illuminated or dark. In the latter case, participants used a flashlight with a diameter of 8$^{\circ }$ visual angle. Importantly, scenes searched with the flashlight were never seen in full light. We implemented the flashlight paradigm^18^ to impede visual search and limit processing of distractors to central vision. After searching, participants were surprised with two subsequent memory tasks, probing identity and location memory of objects, respectively. To test identity memory, participants had to categorize isolated objects in an old/new recognition paradigm as either previously present in one of the scenes (i.e., *old*) or not (i.e., *new*). Location memory was assessed in a scene rebuilding task, in which participants had to place all 20 local objects at the position in the original scene where they had remembered them. First, we conducted the experiment in VR to test the paradigm in a more naturalistic setting where participants could move around in scenes freely and interact with the environment using a handheld controller (Experiment 1). In a separate experiment, we tested the exact same paradigm with different participants on a computer screen in a more classic laboratory setting using only mouse and keyboard as response devices and a chin-rest to control the distance to the screen (Experiment 2). See Fig. 1d for an overview of the experimental procedure. Analyzing both experiments individually but also in combination informs us about how strongly the flashlight manipulation increased search difficulty, how it impacted object memory, and lastly, how robust these elicited effects are across setups.

## Results

### Visual search performance

Searching through dark scenes, only equipped with a flashlight, proved to be more difficult compared to illuminated scenes. As can be seen in Table 1, search accuracy was overall higher in illuminated compared to flashlight scenes (92.38% ± 0.75%; 86.96% ± 0.95%, respectively) but did fail to reach significance in the VR experiment (Illuminated: 91.17% ± 1.16%; Flashlight: 87.83% ± 1.34%; computer screen (CS): Illuminated: 93.48% ± 0.96%; Flashlight: 86.15% ± 1.36%). Importantly, the difference between experiments was not significant.

**Table 1:** Results of the generalized linear mixed-effects model for accuracies of the search task including estimated regression coefficients. *CS* = computer screen, *VR* = virtual reality.

Fixed effects	VR model				CS model			
	Estimate	*SE*	*z*	*p*	Estimate	*SE*	*z*	*p*
Intercept	2.73	0.34	7.93	$<.001$	2.38	0.27	8.83	$<.001$
Condition (Flashlight–Illuminated)	–1.22	0.66	–1.84	.066	–1.02	0.50	–2.02	.043
Trial	0.05	0.04	1.25	.211	0.06	0.04	1.73	.083
Condition $\times$ Trial	0.12	0.08	1.50	.133	0.11	0.07	1.49	.138

**Variance components**	**Variance**	***SD***			**Variance**	***SD***		
Target								
Intercept	1.82	1.35			1.22	1.10		
Condition	1.62	1.27			0.51	0.72		
Participant								
Intercept	0.17	0.41			0.02	0.13		
Condition	0.77	0.88			0.02	0.14		
Pseudo-*R* $^{2}$(*R* $^{2}_{\textrm{marginal}}$, *R* $^{2}_{\textrm{conditional}}$)	.02, .45				.02, .31			

Fixed effects	Full model							
	Estimate	*SE*	*z*	*p*				
Intercept	2.45	0.21	11.57	$<.001$				
Condition (Flashlight–Illuminated)	–0.92	0.38	–2.44	.015				
Trial	0.06	0.03	2.22	.027				
Experiment (CS–VR)	0.04	0.32	0.13	.895				
Condition $\times$ Trial	0.10	0.05	1.98	.048				
Condition $\times$ Experiment	–0.35	0.65	–0.55	.586				
Trial $\times$ Experiment	0.01	0.05	0.14	.888				
Condition $\times$ Trial $\times$ Experiment	–0.02	0.10	–0.16	.873				

**Variance components**	**Variance**	***SD***						
Target								
Intercept	1.28	1.13						
Condition	0.72	0.85						
Participant								
Intercept	0.08	0.29						
Condition	0.31	0.56						
Pseudo-*R* $^{2}$(*R* $^{2}_{\textrm{marginal}}$, *R* $^{2}_{\textrm{conditional}}$)	.02, .34							

**Figure 2:**
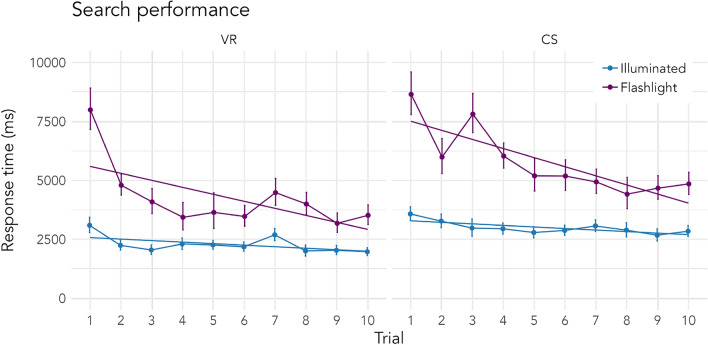
Response times in milliseconds of correct searches from trial 1 to trial 10 within one scene. Solid straight lines represent regression lines. The left panel shows response times of searches in VR, the right panel shows response times of searches in the CS experiment. Solid points indicate means calculated on log-transformed response times, which were converted back to their original form for visualization purposes. Error bars indicate standard errors around the mean. *CS* = computer screen, *VR* = virtual reality.

Besides accuracy, search condition further affected search time. As can be seen in Fig. 2 and Table 2, targets in illuminated scenes were found faster than in flashlight scenes in general (2640 ms ± 368 ms; 4769 ms ± 493 ms, respectively). This effect was also strongly present in both experiments individually (VR: Illuminated: 2302 ms ± 349 ms, Flashlight: 4087 ms ± 502 ms; CS: Illuminated: 2982 ms ± 367 ms, Flashlight: 5513 ms ± 465 ms). Moreover, in both experiments we observed stronger incidental learning effects in the flashlight condition than in the illuminated condition, as indicated by the steeper negative slope across trials (see Fig. 2) and the significant interaction effect of condition and trials. Importantly, this interaction does not differ between experiments as indicated by the non-significant three-way interaction (see Table 2). Overall, participants were slower in the CS experiment, which is most likely due to the somewhat less intuitive handling of mouse and keyboard (VR: 3051 ms ± 454 ms; CS: 3994 ms ± 445 ms). Taken together, we found that the effects exhibited while searching with a flashlight were comparable across VR and computer screen settings.

**Table 2:** Results of the linear mixed-effects model for log-transformed response times during search including estimated regression coefficients.

Fixed effects	VR model				CS model			
	Estimate	*SE*	*t*	*p*	Estimate	*SE*	*t*	*p*
Intercept	8.30	0.06	136.08	$<.001$	8.55	0.06	137.95	$<.001$
Condition (Flashlight–Illuminated)	0.75	0.10	7.58	$<.001$	0.86	0.09	9.30	$<.001$
Trial	–0.05	0.01	–5.89	$<.001$	–0.04	0.01	–5.57	$<.001$
Condition $\times$ Trial	–0.03	0.02	–2.06	.039	–0.04	0.01	–2.83	.005

**Variance components**	**Variance**	***SD***			**Variance**	***SD***		
Target								
Intercept	0.09	0.30			0.09	0.30		
Condition	0.02	0.13			0.03	0.17		
Participant								
Intercept	0.01	0.11			0.02	0.16		
Condition	0.00	0.07			0.01	0.10		
Residual	0.49	0.70			0.44	0.67		
Pseudo$-$*R* $^{2}$(*R* $^{2}_{\textrm{marginal}}$, *R* $^{2}_{\textrm{conditional}}$)	.14, .30				.17, .35			

Fixed effects	Full model							
	Estimate	*SE*	*t*	*p*				
Intercept	8.43	0.05	176.76	$<.001$				
Condition (Flashlight–Illuminated)	0.80	0.07	12.22	$<.001$				
Trial	–0.04	0.01	–8.09	$<.001$				
Experiment (CS–VR)	0.25	0.08	3.21	.002				
Condition $\times$ Trial	–0.04	0.01	–3.47	$<.001$				
Condition $\times$ Experiment	0.11	0.13	0.85	.393				
Trial $\times$ Experiment	0.01	0.01	0.62	.537				
Condition $\times$ Trial $\times$ Experiment	–0.01	0.02	–0.44	.658				

**Variance components**	**Variance**	***SD***						
Target								
Intercept	0.09	0.30						
Condition	0.01	0.08						
Participant								
Intercept	0.02	0.14						
Condition	0.00	0.03						
Residual	0.47	0.69						
Pseudo-*R* $^{2}$(*R* $^{2}_{\textrm{marginal}}$, *R* $^{2}_{\textrm{conditional}}$)	.18, .34							

*CS* = computer screen, *VR* = virtual reality.

### Incidental object identity memory

**Figure 3:**
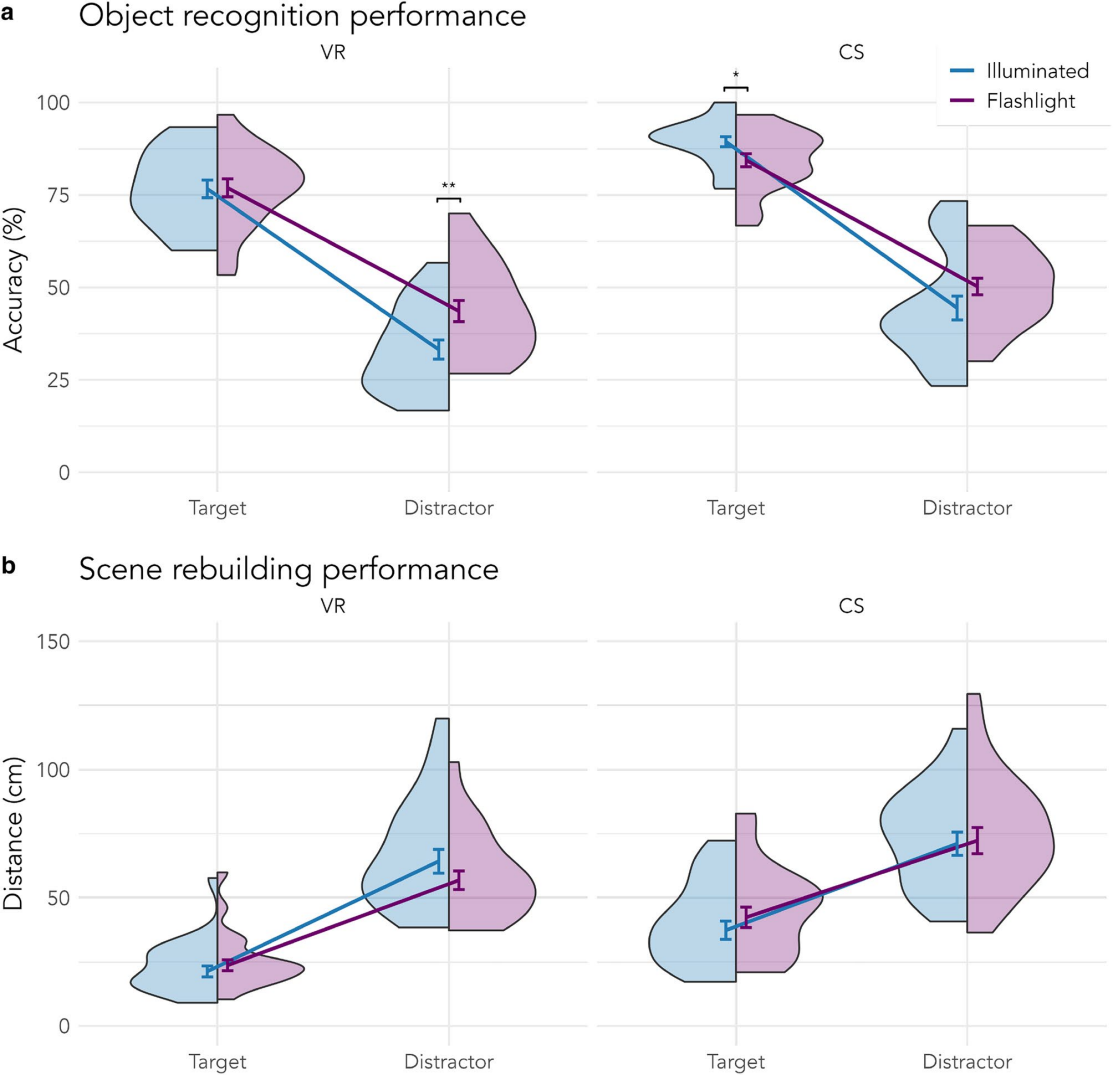
Incidental memory of objects. (**a**) Accuracy in percent of object recognition in the old/new recognition task, (**b**) Distance from the original location in centimeters, calculated on log-transformed distances, which were converted back to their original form for visualization purposes, in the scene rebuilding task. Error bars indicate standard errors around the mean. *CS =* computer screen, *VR* = virtual reality. *p $<.05$; **p $<.01$.

Measuring object recognition with an old/new recognition task allowed us to estimate the incidental memory formation of object identities. As hypothesized, we found an effect of object type, that is, memory recall for targets was higher than for distractors (Targets: 82.04% ± 0.77%; Distractors: 43.01% ± 0.99%). Interestingly, there was no main effect of search condition (Illuminated: 61.18% ± 0.97%; Flashlight: 63.89% ± 0.96%), but an interaction effect between the search condition and the object type. As can be seen in Fig. 3a and Table 3, targets in VR from both conditions were equally well recognized (*b* = −0.01, *SE* = 0.17, *z* = −0.04, *p* = .971), whereas distractors encountered with the flashlight received a significant memory boost (*b* = −0.48, *SE* = 0.15, *z* = −3.22, *p* = .001). In the computer screen experiment, participants showed higher recognition performance in general (VR: 57.58% ± 1.01%; CS: 67.06% ± 0.92%). Interestingly, the flashlight benefit for distractors was also present on-screen, but barely missed significance (*b* = −0.27, *SE* = 0.14, *z* = −1.96, *p* = .050), and contrary to the VR experiment, targets from the illuminated scenes were significantly better recognized than targets from flashlight scenes (*b* = 0.44, *SE* = 0.19, *z* = 2.30, *p* = .022). This indicates that the flashlight search was more difficult on the computer screen than in VR, resulting in a decreased recognition performance for objects encountered during flashlight search.

**Table 3:** Results of the generalized linear mixed-effects model for accuracies of the object recognition task including estimated regression coefficients.

Fixed effects	VR model				CS model			
	Estimate	*SE*	*z*	*p*	Estimate	*SE*	*z*	*p*
Intercept	0.43	0.10	4.21	$<.001$	1.02	0.12	8.75	$<.001$
Condition (Flashlight–Illuminated)	0.26	0.14	1.88	.061	–0.12	0.15	–0.81	.417
Object type (Distractor–Target)	–2.07	0.17	–12.14	$<.001$	–2.26	0.16	–14.25	$<.001$
Condition $\times$ Object type	0.66	0.22	2.99	.003	0.81	0.24	3.36	$<.001$

**Variance components**	**Variance**	***SD***			**Variance**	***SD***		
Object								
Intercept	0.65	0.81			0.56	0.75		
Condition	0.45	0.67			0.25	0.50		
Object type	0.32	0.57			0.18	0.43		
Participant								
Intercept	0.04	0.21			0.11	0.33		
Condition	0.06	0.25			0.15	0.38		
Object type	0.27	0.52			0.16	0.40		
Pseudo-*R* $^{2}$(*R* $^{2}_{\textrm{marginal}}$, *R* $^{2}_{\textrm{conditional}}$)	.21, .39				.24, .40			

Fixed effects	Full model							
	Estimate	*SE*	*z*	*p*				
Intercept	0.72	0.09	8.12	$<.001$				
Condition (Flashlight–Illuminated)	0.08	0.09	0.89	.374				
Object type (Distractor–Target)	–2.12	0.12	–17.58	$<.001$				
Experiment (CS–VR)	0.61	0.11	5.59	$<.001$				
Condition $\times$ Object type	0.59	0.15	3.87	$<.001$				
Condition $\times$ Experiment	–0.33	0.17	–1.92	.055				
Object type $\times$ Experiment	–0.28	0.20	–1.39	.163				
Condition $\times$ Object type $\times$ Experiment	0.23	0.29	0.80	.424				

**Variance components**	**Variance**	***SD***						
Object								
Intercept	0.55	0.74						
Condition	0.03	0.18						
Object type	0.37	0.61						
Participant								
Intercept	0.07	0.27						
Condition	0.09	0.29						
Object type	0.21	0.46						
Pseudo-*R* $^{2}$(*R* $^{2}_{\textrm{marginal}}$, *R* $^{2}_{\textrm{conditional}}$)	.24, .39							

*CS* = computer screen, *VR* = virtual reality.

### Incidental object location memory

**Figure 4:**
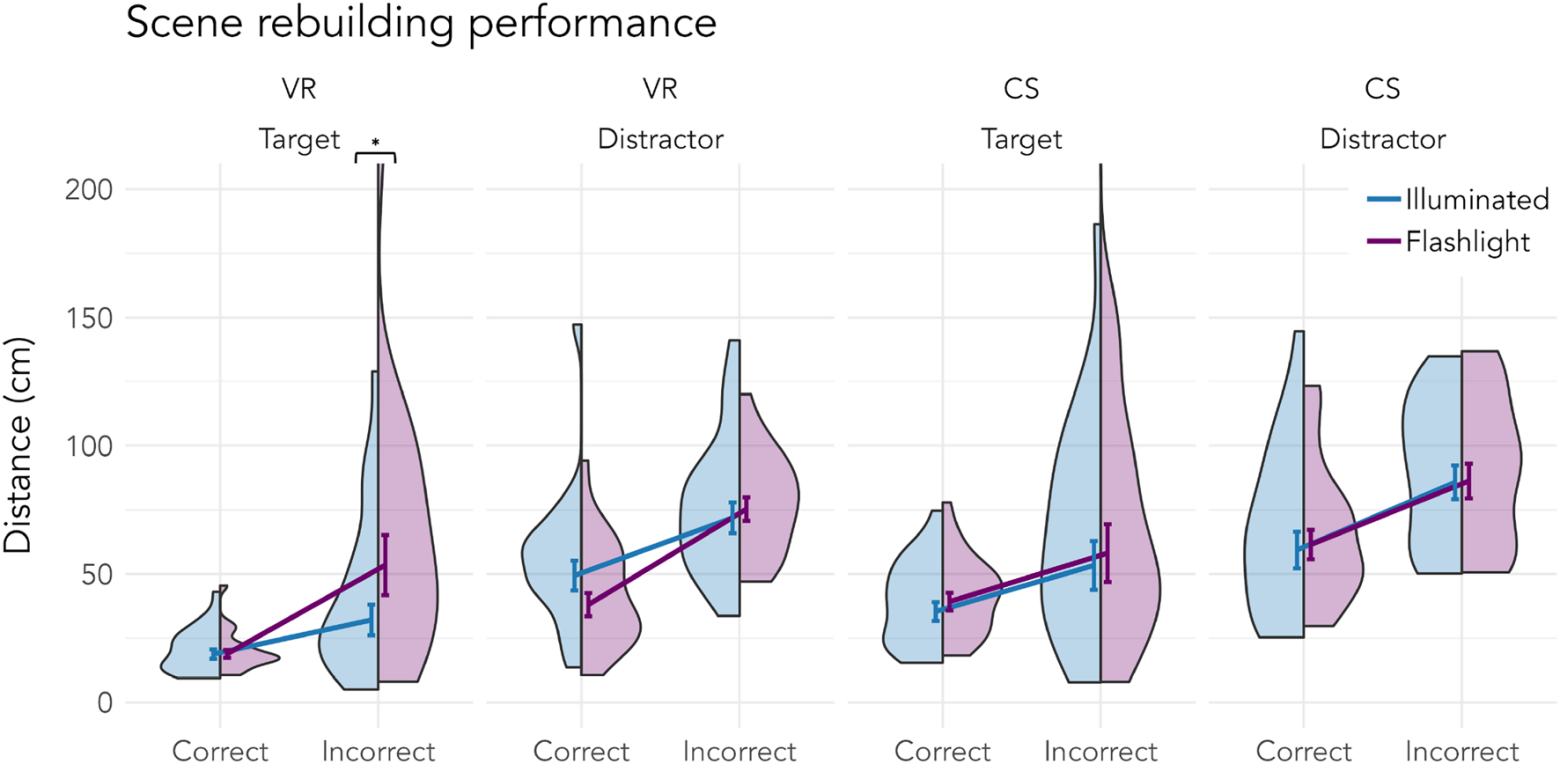
Incidental location memory of objects split into correct and incorrect recognition in the object recognition task. Distance from the original location in centimeters in the scene rebuilding task. Error bars indicate standard errors around the mean calculated on log-transformed distances, which were converted back to their original form for visualization purposes. Note that the figure is truncated at 200 cm and does not display three data points as indicated by the two violin plots reaching the upper bound. The full figure can be found in the online supplementary material. *CS* = computer screen, *VR* = virtual reality. *p $<.05$.

Lastly, we were interested in how well participants remembered not only objects’ identities but also their locations. Again, we found a benefit for actively searched objects with targets being placed closer to their original locations than distractors (Targets: 29.88 cm ± 9.53 cm; Distractors: 66.00 cm ± 9.25 cm). In general, objects were placed with higher precision in the VR than in the computer screen experiment (VR: 36.82 cm ± 10.44 cm; CS: 52.94 cm ± 8.81 cm). This is, again, probably due to the less intuitive object placement via mouse and keyboard and the lack of depth perception on the computer screen. Interestingly, we found an interaction between search condition and object type. Looking at Fig. 3b and Table 4, it becomes evident that this interaction was only present in the VR experiment, due to the worse positioning of distractors encountered in illuminated scenes. However, none of the post-hoc comparisons were significant (VR: Target *b* = −0.11, *SE* = 0.09, *z* = −1.16, *p* = .245, Distractor *b* = 0.09, *SE* = 0.09, *z* = 1.03, *p* = .305; CS: Target *b* = −0.12, *SE* = 0.09, *z* = −1.38, *p* = .169, Distractor *b* = −0.03, *SE* = 0.09, *z* = −0.36, *p* = .716). Although the main effect of object type shows that targets were better memorized in terms of identity and location, it is indeterminable whether there was any memory for distractors or if performance was simply at chance level. The chance level is difficult to determine since all scenes were semantically and syntactically coherently furnished and thus participants’ distractor performance could simply reflect the performance of pure guessing in combination with the application of their scene grammar knowledge^38^. To get a better insight into actual incidental location memory, we further split up the rebuilding data by including the accuracy of the object recognition task. Objects which were correctly recognized as present in the scenes might also be better remembered spatially. Therefore, we reran the last analysis with recognition accuracy as an additional fixed effect which was allowed to interact with the other fixed effects (see formula 5). The result table and follow-up post-hoc tests can be found in the online supplementary material. The overall results of the LMM are comparable to the results in Table 4 with an additional highly significant main effect of previous recognition accuracy. This indicates that identity and location memory were indeed encoded in conjunction. Follow-up post-hoc tests further established that within each experiment, each of the comparisons of recognition accuracy between search condition and object type was significant (all *p*s $<.037$). In other words, correctly recognized objects in the object recognition task were also positioned closer to the original location in the scene rebuilding task. This was true for all object types (targets as well as distractors) in both conditions (illuminated and flashlight) and both experiments (VR and computer screen). We were further interested in the effect of the flashlight paradigm and computed the corresponding post-hoc tests. Looking at Fig. 4, only in the VR experiment did the search condition lead to differences. Previously incorrectly recognized targets from the flashlight conditions were also positioned worse compared to the illuminated condition (*b* = −0.46, *SE* = 0.15, *z* = −3.00, *p* = .003), whereas previously correctly recognized distractors from the flashlight condition were descriptively but not significantly better positioned than those from the illuminated condition (*b* = 0.22, *SE* = 0.12, *z* = 1.77, *p* = .078), resembling the pattern found in the object recognition task. All other comparisons were not significant (all *p*s $>.221$).

**Table 4:** Results of the linear mixed-effects model for log-transformed distances of placed objects in the scene rebuilding task including estimated regression coefficients.

Fixed effects	VR model				CS model			
	Estimate	*SE*	*t*	*p*	Estimate	*SE*	*t*	*p*
Intercept	−1.00	0.07	−13.38	$<.001$	−0.63	0.07	−8.41	$<.001$
Condition (Flashlight–Illuminated)	−0.01	0.10	−0.08	.938	0.07	0.04	1.59	.128
Object type (Distractor–Target)	0.98	0.05	18.38	$<.001$	0.61	0.04	13.64	$<.001$
Condition $\times$ Object type	−0.21	0.11	−1.99	.047	−0.09	0.09	−0.95	.341

**Variance components**	**Variance**	***SD***			**Variance**	***SD***		
Object								
Intercept	0.34	0.59			0.28	0.53		
Participant								
Intercept	0.04	0.20			0.06	0.25		
Condition	0.13	0.36			0.00	0.02		
Residual	1.63	1.28			1.20	1.10		
Pseudo-*R* $^{2}$(*R* $^{2}_{\textrm{marginal}}$, *R* $^{2}_{\textrm{conditional}}$)	.11, .29				.06, .27			

Fixed effects	Full model							
	Estimate	*SE*	*t*	*p*				
Intercept	−0.81	0.06	−12.93	$<.001$				
Condition (Flashlight–Illuminated)	0.04	0.05	0.78	.440				
Object type (Distractor–Target)	0.80	0.03	22.96	$<.001$				
Experiment (CS–VR)	0.37	0.08	4.80	$<.001$				
Condition $\times$ Object type	−0.14	0.07	−2.07	.039				
Condition $\times$ Experiment	0.07	0.11	0.66	.512				
Object type $\times$ Experiment	−0.36	0.07	−5.17	$<.001$				
Condition $\times$ Object type $\times$ Experiment	0.11	0.14	0.80	.425				

**Variance components**	**Variance**	***SD***						
Object								
Intercept	0.29	0.54						
Participant								
Intercept	0.05	0.23						
Condition	0.07	0.26						
Residual	1.43	1.20						
Pseudo-*R* $^{2}$(*R* $^{2}_{\textrm{marginal}}$, *R* $^{2}_{\textrm{conditional}}$)	.10, .28							

*CS* = computer screen, *VR* = virtual reality.

## Discussion

In this study, we tested the effects of a naturalistic flashlight paradigm on visual search performance and incidental identity and location memory formation. To this end, we conducted two experiments which were equal in their administered tasks but differed in the way they were presented to the participants: in VR or on a computer screen. In both experiments, participants had to successively search for objects within three-dimensional indoor scenes. They completed the searches either in full light or in darkness, equipped with a flashlight to only reveal part of the field of view. Afterwards, participants were surprised with two memory tasks (i.e., object recognition task and scene rebuilding task), probing their incidental memory of object identities and locations built during search. Importantly, participants fulfilled the tasks either immersed in VR with a handheld controller or seated in front of a computer screen with a chin-rest, which required the handling of navigation through the three-dimensional environment via mouse and keyboard. Due to recent technical developments in VR, it is now becoming increasingly affordable and feasible to implement paradigms in VR and thereby place participants in immersive, actionable environments. Thus, we were also interested in the generalizability of our new flashlight paradigm across virtual and computer screen modalities.

We found that the flashlight manipulation did—as intended and similar to designs using gaze-contingent windows^19–21,39,40^—increase search difficulty in both the VR and the computer screen experiment. Search patterns were comparable across experiments, apart from the fact that search times were overall faster in VR. This difference might stem from the more intuitive handling using a controller and changing visual input by turning one’s head and body compared to using a mouse and a keyboard while remaining seated and stationary. Additionally, the computer screen could only display a smaller area of the scene excluding visual peripheral information which might have benefitted search in VR. Absolute search times in computer screen studies using virtual environments should thus not be interpreted at face value.

With regards to object memory, we found that previously searched targets were better remembered in terms of identity and position than distractors, regardless of whether the view was obstructed during search or whether the search took place immersed in a VR environment or on a computer screen. We hypothesized that searching with a flashlight has an effect on object memory of distractors but did not specify the direction of the effect a priori. Either distractors might be remembered worse: Increased search difficulty might take a toll on incidental memory and some distractors might never be seen because they never came into the flashlight window, a possibility in the experiment that could not be controlled. On the other hand, there were reasons to assume better incidental memory for distractors: Increased search difficulty has been shown to lead to better memory performance in the past^32,41^ and the limited visual input could lead to more elaborate foveal processing^18,19^. Here, we found a memory benefit for distractors encountered in dark scenes in the VR experiment, whereas performance for targets was comparable. In the computer screen experiment, we found a similar benefit for distractors from the flashlight condition, but it did not reach statistical significance. Interestingly, in the computer screen experiment, targets from the illuminated condition were recognized significantly better than from the flashlight condition. Given the absence of this pattern in the VR experiment, this could indicate that the increased difficulty of the flashlight paradigm was higher in the computer screen experiment. Although searching with a flashlight in VR might have been experienced as natural enough to not affect the benefit of active searching, it did cause stronger interference in the computer screen paradigm, resulting in an overall decreased memory for objects encountered in the dark.

Following the object recognition task, we assessed participants’ incidental location memory with a scene rebuilding task. Surprisingly, we found no differences between search conditions, indicating that objects in the flashlight condition were positioned as accurately as those in the illuminated condition, despite participants never having seen the full, illuminated scene. Even though participants did not process the scene in its entirety, they were still able to position the objects as accurately as when they had encoded the fully visible scene. This hints at the human brain’s ability to create spatial layout representations by piecing together incoming fragments in succession. Although surprising, our findings are in line with previous research showing that spatial updating is a highly robust, automatized, and reflex-like cognitive mechanism, which works remarkably well under visual or actionable constraints (e.g., limited visual input or viewing another person navigating on a computer screen, respectively)^42–44^. An alternative explanation, which we cannot rule out, could be that the limited visual information available through the flashlight’s 8$^{\circ }$ window was sufficient for participants to incidentally learn an object’s precise location and context. However, this seems unlikely given that the window’s diameter was deliberately sized to correspond with the dimensions of most local objects, allowing typically only one local object to be visible at a time and making the spatial relationships between objects, which are crucial for accurate rebuilding, almost imperceptible. For instance, knowing that a water bottle is on a kitchen counter does not imply that it can be placed correctly (i.e., in the correct spot on the counter) if the location of other objects on the exact same counter, like a banana or a coffee mug, are unknown.

To better understand the actual effect of memory on performance, we further included the accuracy data of the object recognition task in the analysis of the rebuilding data. As expected, we found that object recognition performance positively predicted scene rebuilding performance: Objects that had previously been recognized accurately were also positioned closer to their original locations. This indicates that identity and location memory were incidentally formed in conjunction^3,45^, which is in line with past research observing the same relationship in a real-world environment^12^. Interestingly, when accounting for object recognition accuracy, we found an interaction between object type and search condition only in the VR experiment but not in the computer screen experiment. Specifically, targets that were not recognized in the object recognition task were placed further from their original position in dark scenes compared to those in illuminated scenes. Conversely, distractors from dark scenes that were correctly recognized were also positioned more accurately, although this difference did not reach statistical significance when compared to correctly recognized distractors in illuminated scenes. This indicates that searching with a flashlight in the dark put a toll on the memory boost of actively searched targets, while it increased memory usage as shown by higher incidental memory for distractors. These effects were absent in the computer screen experiment. The presence of these effects in the VR experiment could be explained by a sensorimotor spatial encoding benefit. That is, while spatial encoding also works when perceiving actions passively through visual input, the system works more precisely when the visual input is actively generated by self-coordinated actions from an egocentric frame of reference^42,43,46,47^. Even the visibility of one’s own limbs is necessary to achieve an optimally working process^48^. On the other hand, the system is rather robust: Actions can be performed successfully even without visual input^49^, and gaps in encoding can be filled in through cognitive inference^50^. In our experiment, the benefit of sensorimotor encoding for spatial representations might explain the small differences we found in the rebuilding task but the lack of any major differences overall. It appears that such possible differences between setups may be a lot smaller and would need a higher sample size to be reliably detected. Further investigations with larger samples are essential to validate and expand upon our findings, particularly in exploring the nuanced differences in cognitive processing across VR and 3D computer screen environments. These differences might shed further light on how human cognition shapes action in daily-life surroundings. Once the existence of potential differences between immersive VR and dynamic 3D screen environments has been better established, future research may look deeper into the differences between 3D and static 2D environments, which could imply meaningful differences in cognitive processes active in the real world versus in the “psychologist’s laboratory”^24^. As technology advances, the question arises: To what extent can virtual experiences emulate the complexities of human cognition in the real world, and how might this reshape our approach to the “real-world or the lab”-dilemma^24^?

The use of a handheld flashlight in VR could potentially elicit highly naturalistic eye and hand movements, closely mimicking the coordination required in real-world tasks. As our study lacks eye-movement data, future studies might want to record eye- and hand-movement data to complement the picture. Given the smaller field of view of the computer screen and the flashlight, it could be that participants exhibit smaller and fewer eye movements overall. The visual information provided by the flashlight window on the 2D screen could be processed with high visual acuity and minimal need for eye movements whereas this is not feasible in VR, where the flashlight window extends beyond the fovea. In this context, it would be interesting to study possible differences in eye-movement patterns between VR and on-screen experiments as a function of limited visual input. Moreover, it could be worthwhile to investigate whether differences in memory between experiments remain when using gaze-contingent windows, which feel unnatural in both VR and computer-based studies. Such research could provide insights into the nuances of hand-eye coordination, particularly focusing on how this interaction adapts when participants are required to learn and adjust to a novel control mechanism like a gaze-contingent window. This could further illuminate the dynamic interplay between visual attention and motor control in interactive environments.

Our findings also invite further investigation into how different types of contingent-window paradigms may differentially affect cognitive processes. While both gaze-contingent and hand-contingent windows affect visual search efficiency, the impact of our flashlight paradigm on eye movements is unclear. A thorough comparison of these effects, however, would necessitate a nuanced analysis incorporating hand-movement data to parallel the eye-tracking measures commonly assessed in gaze-contingent studies^19–21^. We thus propose that future research should delve deeper into these comparisons to unravel the intricate relationship between visual search impairments, hand- and eye-movement dynamics, and the type of contingent window used, whether it be gaze-, mouse- or controller-contingent.

Lastly, one factor that potentially impacted memory performance in our design was the increased risk of unseen objects during flashlight search. While objects in the illuminated condition which were not fixated could still be encoded into memory through peripheral vision, the 8$^{\circ }$ diameter of the flashlight window truly limited visual processing to the central foveal area. Consequently, objects were only foveally processed if they were within the flashlight window and any objects outside of this window remained unseen. As unseen objects cannot be memorized, they naturally could not be recalled during the surprise memory tasks, and in turn may have negatively impacted aggregate measures of memory performance. By tracking which objects were visible to the participant through the flashlight window during search, we could assess that during flashlight search in VR, 95.84% (*SD* = 3.22%) of targets and 79.47% (7.65%) of distractors were seen, while on computer screen, 95.61% (3.76%) of targets and 74.39% (10.81%) of distractors were seen. This confirms that the vast majority of targets and a substantial proportion of distractors were indeed seen during flashlight search. However, the absence of eye-tracking data for the illuminated condition precludes a direct comparison of object visibility between the two search conditions. Consequently, comparing only objects seen in the flashlight condition with all objects in the illuminated scenes introduces a bias, as it includes potentially also unseen objects in the latter. This methodological constraint highlights the need for future studies to incorporate comprehensive eye-tracking measures. Including measures known to correlate with memory performance, such as summed gaze duration per object^10,12,18,51^, will prove advantageous in further disentangling the complex interplay between visual search, task difficulty, and incidental memory.

## Conclusion

Our study tested the feasibility of using a naturalistic flashlight paradigm to simulate an ecologically valid type of limited visual input during visual search through scenes and how it affects incidental memory formation for object identities and locations. In particular, we implemented the same study in both immersive VR and dynamic 3D computer screen setups. Across experiments, we found that searching through dark scenes using a flashlight, as opposed to fully illuminated scenes, increased search difficulty and influenced object memory, with observable impacts on identity memory. Location memory showed comparable results initially, but further analyses accounting for object recognition performance revealed differences in the VR experiment. These findings suggest potential differences in cognitive processing in VR compared to the computer screen experiment, which may be due to the immersive nature of VR, resulting in an interaction of actions and incidental memory formations. Moreover, we replicated previous findings^6,11,12,14^ showing that memory was increased for objects which were previously searched compared to objects which were simply present in the scenes. Surprisingly, we found that removing visual information did not necessarily compromise location memory. These findings not only reaffirm the adaptability of human memory processes in diverse environments but also open new avenues for exploring how technological advancements like VR can mimic and extend our understanding of cognitive processes in real-world settings. Importantly, our study demonstrates that screen experiments using virtual environments can elicit the same major effects as VR setups. Thus, virtual environments on traditional computer screens can also be effectively used for research aiming for ecological validity, offering a more feasible alternative to the often complex and resource-intensive full-fledged VR setups.

## Methods

### Participants

Based on sample sizes of previous research in the field^11,13–15,29,32,33^, we aimed to collect data of 20 participants per experiment. In Experiment 2, we had to exclude single trials of two participants. One participant aborted the experiment due to nausea during the final rebuilding task, leaving two scenes not rebuilt (33.33% of rebuild trials of the participant) and left out of further analyses. Another participant saw one scene flipped upside down during flashlight search due to a computer error. This rendered the task increasingly more difficult, so we excluded the affected search trials (16.67% of search trials) as well as the trials of the recognition task (8.33%) and the scene of the rebuilding task (16.67%). Since we could not use the full data of these two participants, we collected two additional participants, rendering our final sample sizes 20 and 22, respectively (Exp1: mean age = 20.30 years, range = 18–27 years, 16 women, four men; Exp2: mean age = 23.78 years, range = 19–36 years, 13 women, nine men). All participants had normal or corrected-to-normal vision (in Experiment 1, only contact lenses were allowed), were tested for visual acuity (at least 20/25) and normal color vision as assessed by the Ishihara test. All participants spoke German at native level, were naïve to the hypotheses and the stimuli except for two participants of Experiment 2, who were exposed to some of the stimuli two years before in a 2D computer experiment. All participants were recruited at Goethe University Frankfurt, volunteered, gave informed consent, and were compensated with either course credit or 10 €/h. The experimental procedure conformed to the Declaration of Helsinki and was approved by the local ethics committee of the Faculty of Psychology and Sports Sciences (2014-106R1) at Goethe University Frankfurt.

### Apparatus

In Experiment 1, participants wore an HTC Vive head-mounted display (HMD) and held an HTC Vive controller in their dominant hand (see Fig. 1b). The HMD comprised two OLED screens with dimensions of 1080 $\times$ 1200 px, a refresh rate of 90 Hz, and a combined field of view (FoV) of approximately 100$^{\circ }$ (horizontally) $\times$ 110$^{\circ }$ (vertically). The diameter of the flashlight window had a constant size of 8$^{\circ }$ of visual angle. The experimental procedure was implemented in C# in the Unity 3D game engine (version 2017.3) using SteamVR (version 1.10.26) on a computer running Windows 10.

In Experiment 2, the experiment was executed on the same computer as in Experiment 1 but now scenes were displayed on a 22-inch monitor with a resolution of 1680 $\times$ 1050 px and a refresh rate of 60 Hz (see Fig. 1b). In computer graphics, a virtual camera with certain projection parameters is set to render a virtual scene. These parameters will determine the FoV of the camera and thus how much of the virtual world will be rendered on screen. On traditional desktop displays, the FoV is approximately 60$^{\circ }$ vertically, the horizontal FoV is usually determined by the screen’s aspect ratio. It should be understood that in this case a camera’s FoV is not the same as the size of the display in visual angles in a viewer’s FoV. However, in VR, for the purpose of immersiveness the virtual camera’s FoV will approach the viewer’s FoV. In Experiment 2, viewing distance was 60 cm ensured by a chin-rest, resulting in a FoV of 43.27$^{\circ }$ (horizontally) $\times$ 25.18$^{\circ }$ (vertically) on participants’ retina, while the Unity virtual camera rendered the virtual environment with a FoV of 91.5$^{\circ }$ (horizontally) $\times$ 60$^{\circ }$ (vertically). While the diameter of the flashlight window had the same size of 8$^{\circ }$ of visual angle in the virtual environment and thus revealed equally much visual information as the flashlight in VR, the flashlight window in the participants’ FoV was closer to 3.5$^{\circ }$.

### Stimuli

In both experiments, we used the same set of in-house developed indoor scenes also used in previous studies^11,18,19,21,29,52^. Here, we selected six scenes, two each from three different room categories: bathroom, kitchen, and living room (see Fig. 1a). Every scene spanned approximately 380 $\times$ 350 $\times$ 260 cm (length $\times$ width $\times$ height), which was fitted to the room size of the laboratory where Experiment 1 took place so that participants could naturally move in virtual rooms without fear of colliding with walls. Each scene contained eight large, static objects (also known as global objects or anchor objects^38,52,53^, e.g., sink, refrigerator, bed) and 20 smaller, local objects, which are often interacted with (e.g., toothpaste, pan, alarm clock). These local objects were used as target and distractor objects. An additional gray room was used for practice trials. This training scene included three global and 10 local objects which were considered uncommon for typical indoor living spaces (e.g., hydrant, traffic light, stethoscope) to avoid any interference such as priming of any of the succeeding scenes of the actual search task. The same practice room was also used in Experiment 2 to practice rebuilding. The object recognition task consisted of the 120 local objects encountered during search and an additional 120 new local objects, which were of comparable type and used as lures.

### Design

In both experiments we implemented the exact same experimental routine, that is, a repeated visual search task followed by a surprise object recognition task and a surprise scene rebuilding task. In the visual search task, we manipulated search difficulty by using a flashlight paradigm^18^, which implements a controller-contingent window, intuitively similar to using a flashlight. In Experiment 1 in VR, a pink circle was placed in the center of the flashlight window. In illuminated searches, a pink laser beam emerged from the controller which had to be placed on the targets. Importantly, in Experiment 2 on computer screen, the flashlight window was fixed to the center of the screen with a fixed pink circle in its center. Fully illuminated scenes only showed the pink circle. See Fig. 1c for example images displaying the conditions. Conditions (flashlight vs. illuminated) were presented in blocks and the order of blocks was balanced across participants. Each block was preceded by practice trials to get familiar with the condition. Both conditions contained three randomly chosen scenes with each of the three scene categories (i.e., living room, bathroom, and kitchen; see Fig. 1a) present once per condition. The condition in which a scene appeared was balanced across participants. For each participant individually, 10 out of the 20 local objects were randomly chosen as targets, defining the other 10 as distractors. The search order of objects was randomized. Each scene appeared only once and all 10 target objects were searched in immediate succession. Participants completed a total of 60 searches.

The visual search task was followed by a surprise object recognition task to test incidental memory, in which participants had to decide if an object was old (i.e., present in one of the searched scenes) or new. All 120 local objects of the scenes and an additional 120 new objects were presented (i.e., 240 objects in total). Thus, chance level was at 50%. Trial order was randomized. In Experiment 2, participants had to additionally rate their confidence of their decision on a scale from 1 (not confident at all) to 6 (very confident). The confidence ratings data are not analyzed here, but are published online with the rest of the data.

Lastly, participants performed a scene rebuilding task, in which all six previously searched scenes had to be rebuilt from memory. Crucially, all large global objects were already at their original position and only the 20 local objects were on a pile and had to be placed, resulting in 120 objects which needed to be positioned. A video of the tasks in both experiments can be found here: doi.org/10.17605/osf.io/8bqya.

### Procedure

#### Experiment 1

Upon arrival, participants gave informed consent and performed both vision tests. Participants were then familiarized with the HMD and how to use the VR controller. Regarding the visual search task, participants were told to search as fast and precisely as possible, that each object to be searched is always present in the scene, and that they could navigate freely within the virtual room while searching. No information regarding strategies or subsequent testing of memory was given. Prior to the actual start of the task, participants performed practice trials in a gray neutral room. Search conditions were in blocks and before each block, participants practiced to get familiar with the condition. Before entering a scene, participants were in an empty gray room with instructions written on a wall. Participants then had to position themselves on a blue square on the floor, which was the starting position for the scene and from where they would see most of the objects without obstructions. When participants were ready, they pulled the trigger button on the VR controller to start the trials. In the scenes, the blue squares were not visible. When search trials in a scene started, a fixation cross appeared on a large black square in the center of the participants’ visual field of view for 1 s, followed by a verbal target cue for 1.5 s that informed participants which object to search for (e.g., “Badeente”, rubber duck in German). When the cue disappeared, participants had 15 s to find the target in the illuminated condition and 30 s in the flashlight condition. Participants completed the trial by pointing a laser beam emerging from their controller in the illuminated condition or placing the pink circle in the center of the flashlight window at the target. They then completed the trial by pulling the controller’s trigger with their index finger. In case the selected object was not the target or the timeout was reached (i.e., 15 s and 30 s for the illuminated and flashlight condition, respectively), participants heard an error sound and the trial was scored as incorrect. Upon pulling the trigger or after the timeout, the fixation cross cueing the next search appeared. After searching for all 10 target objects successively, participants again entered a gray room with instructions on the wall and continued with the next scene. After finishing the search task (i.e., both the illuminated and flashlight block), participants could take a brief break.

They then continued with the object recognition task. Here, participants were standing in a gray empty room. In the center of the room, a local object was floating mid-air and participants had to decide whether the object is old (i.e., it was present in one of the searched scenes either as a target or distractor) or new (i.e., entirely new, never seen before and was not present in the scenes searched before). They indicated their decision by pulling the trigger button with their index finger or pressing the touch pad with their thumb for old or new, respectively. No timeout was specified, but participants were instructed to decide intuitively and not think about it too much. Participants did not receive any feedback. After making a decision, the object disappeared and 0.5 s later, the next object appeared at the same position. After finishing the object recognition task, participants could again take a brief break and take off the HMD.

Lastly, participants performed the scene rebuilding task. Prior to starting the actual task, participants were familiarized with the handling and placing of objects. To move an object, they would intersect the object with the controller and then pull the trigger to grab the object, move it with the controller while keeping the trigger pulled, and eventually let it go by releasing the trigger. Independent of the search condition, all scenes were fully illuminated during rebuilding, and all global objects were already in place with all 20 local objects lying on a pile to be placed. Participants were instructed to place all objects where they remembered them. For each scene, participants were given 5 minutes to refurnish the scene until they were confident or until timeout. In case participants finished early, they could end the rebuilding of the scene by pushing the menu button of the VR controller. If the timeout was reached and participants were still holding an object, time continued until the object was placed. The remaining time was always displayed on the controller. If needed, participants could take breaks between scenes. The entire procedure including all three tasks, as depicted in Figure 1d, took approximately 1.5 h.

#### Experiment 2

In Experiment 2 (CS), participants followed the same procedure as in Experiment 1 (VR) but with slight adjustments made to accommodate for a desktop-computer screen. Before starting the experiment, participants adjusted the chin-rest to a comfortable height. Navigation in Experiment 2 emulated a first-person-perspective computer game, that is, participants moved through the virtual environment by using the arrow keys forward, backward, left, and right for translations of the camera, as well as the computer mouse to control the rotation of the camera. Similarly to computer games, the pink circle acting as a cursor was fixed to the center of the screen. Due to this technical implementation, the flashlight window was similarly fixed to the center. Importantly, the exact same 3D virtual environments as in Experiment 1 were used and participants could navigate through them as in VR. When having found the target, participants had to place the pink circle on it and log their response by pressing the left mouse button. In case of an error, the frame of the screen flashed red for 1 s.

In the object recognition task, the camera view was stationary and participants had to press the *A* or *N* key on the keyboard for old and new (in German *alt* and *neu*), respectively. After each decision, participants had to rate their confidence on a scale from 1 (not confident at all) to 6 (very confident).

Lastly, participants engaged in the scene rebuilding task. Navigating the 3D scenes was identical to the search task, that is, participants navigated the virtual environments using the arrow keys and the mouse. Objects could be picked up by placing the pink circle on the objects and pressing the left mouse button. In addition to the navigation, there were now two more options on how to handle the objects: Objects could be rotated along their local vertical (*W* and *S* keys) and horizontal axis (*A* and *D* keys), and could be moved closer to or further away from the camera using the mouse wheel. Due to the more unnatural handling with mouse and keyboard, participants in Experiment 2 received additional training for this task to ensure that they were capable of completing it. The experimenter instructed participants and gave them feedback while they were rebuilding the practice room. Inside the practice room, the original object locations were highlighted with a light green silhouette of each object, providing information on where the object was placed, so that participants could focus on learning the technical handling during practice. Once position and rotation fitted with a maximum deviation of 10$^{\circ }$, the object snapped into place, becoming immovable, thus informing the participants that objects did not need to be placed perfectly accurately but more or less into the right position. The training was not timed, ensuring that each participant could take the time they needed to acquire the skills. Once all 10 practice objects were placed, participants started the actual task. A timer was counting down in the corner of the screen. Participants were instructed to focus on location rather than rotation and to try to place as many objects as possible, preferably all. The entire procedure including all three tasks took approximately 2 h.

### Data analysis

For analyzing response times of the search task, only correct trials were included, which resulted in the removal of 259 trials (i.e., 10.28% of the complete data including both experiments). No trials from the object recognition task were excluded. In the scene rebuilding task, only objects which were never moved were excluded, resulting in the removal of 113 trials (2.24%) from Experiment 2 and none from Experiment 1. As mentioned in the Participants section, for one participant in Experiment 2, one scene was flipped upside down, which resulted in the removal of 10 search trials (0.40%) as well as the accompanying 20 objects of the recognition task (0.20%) and the 20 objects of the scene rebuilding task (0.40%). Another participant aborted the experiment after rebuilding the third scene, leading to an exclusion of 40 objects of the scene rebuilding task (0.80%). The final data set consisted of 2510 search trials, 5020 object recognition trials, and 4867 placed objects in the scene rebuilding task.

We analyzed our data using the R statistical programming language (version 4.3.0^54^) with RStudio (version 2023.6.1.524^55^). To investigate search behavior and memory performance, we calculated generalized linear mixed-effects models (GLMMs) and linear mixed-effects models (LMMs) on all variables of interest using the lme4 package (version 1.1-32^56^). Performing a mixed-model approach allowed us to estimate and account for both between-subject and between-stimulus variance simultaneously, which is advantageous compared to traditional F1/F2 analyses of variance^57,58^. We analyzed search accuracy, search time, object recognition accuracy, and scene rebuilding accuracy measured as distance to the original location. Variables of interest analyzed with LMMs (i.e., search time and scene building accuracy) were log-transformed to meet assumptions and approximate a normal distribution of the residuals. Both search accuracy and object recognition accuracy were modeled with GLMMs using a binomial distribution. In all our full models, we included experiment (i.e., VR and CS) and search condition (i.e., Illuminated or Flashlight) as fixed effects. The search time and search accuracy models further included a fixed effect for trial number, and both the object recognition accuracy model and the scene rebuilding accuracy model included a fixed effect for object type (i.e., Target and Distractor). To derive the optimal random effects structure, we started with the maximal effects structure including random intercepts and random slopes for all fixed effects for both participant and object, and subsequently reduced the model to ensure convergence and a non-singular boundary fit^59^. The best-fitting model was assured by comparing competing models with the Akaike Information Criterion^60^. We report the final model structures in Wilkinson notation^61^ in formulae 1-5. To directly compare search condition (Illuminated vs. Flashlight), object type (Target vs. Distractor), and experiment (VR vs. CS), we implemented difference contrasts. Separate models and contrasts for the VR and computer screen experiment consisted of the same effects structure only without the fixed between-effect of experiment. We obtained *p*-values for LMMs by estimating degrees of freedom with the Satterthwaite method provided by the lmerTest package (version 3.1-3^62^). *p*-values for GLMMs were based on asymptotic Wald tests from the lme4 package (version 1.1-32^56^). All models were fitted with the restricted maximum-likelihood criterion. For each model, we report unstandardized regression coefficients with the *t* or *z* statistic (for LMMs or GLMMs, respectively), and the results of a two-tailed test corresponding to a 5% significance level, and Pseudo-*R*^2^-estimates^63,64^ derived with the package MuMIn (version 1.47.5^65^). Post-hoc tests for the object recognition and scene rebuilding task are reported with a Bonferroni correction and were computed on the full model. Figures were created with the ggplot2 package (version 3.4.2^66^). Dependent measure variable means are reported with their standard error (i.e., $M \pm SE$), and variables which were log-transformed (i.e., search time and distance) for modeling are reported as back-transformed summary statistics.

Model search accuracy

1 $$\begin{aligned} Accuracy&\sim 1 + SearchCondition * TrialNumber * Experiment\, \nonumber \\&\quad +(1 + SearchCondition\, |\, Participant)\, \nonumber \\&\quad +(1 + SearchCondition\, |\, Target) \end{aligned}$$

Model search time

2 $$\begin{aligned} log(SearchTime)&\sim 1 + SearchCondition * TrialNumber * Experiment\, \nonumber \\&\quad +(1 + SearchCondition\, |\, Participant)\, \nonumber \\&\quad +(1 + SearchCondition\, |\, Target) \end{aligned}$$

Model object recognition accuracy

3 $$\begin{aligned} Accuracy&\sim 1 + SearchCondition * ObjectType * Experiment\, \nonumber \\&\quad +(1 + SearchCondition + ObjectType\, |\, Participant)\, \nonumber \\&\quad +(1 + SearchCondition + ObjectType\, |\, Object) \end{aligned}$$

Model scene rebuilding accuracy

4 $$\begin{aligned} log(Distance)&\sim 1 + SearchCondition * ObjectType * Experiment\, \nonumber \\&\quad +(1 + SearchCondition\, |\, Participant)\, \nonumber \\&\quad +(1 \, |\, Object) \end{aligned}$$

Model scene rebuilding accuracy accounting for object recognition

5 $$\begin{aligned} log(Distance)&\sim 1 + SearchCondition * ObjectType * RecognitionAccuracy * Experiment\, \nonumber \\&\quad +(1 + SearchCondition\, |\, Participant)\, \nonumber \\&\quad +(1 \, |\, Object) \end{aligned}$$

## Supplementary Information

Supplementary Information.

## Data Availability

The presented study was not preregistered. Preprocessed data, the corresponding analysis script, online supplementary materials, and videos of the tasks of both experiments are available on the Open Science Framework and can be accessed at doi.org/10.17605/osf.io/8bqya.
