# Supplementary material for: Using a flashlight-contingent window paradigm to investigate visual search and object memory in virtual reality and on computer screens
Source: Sci Rep. 2024 Apr 13;14:8596. doi: 10.1038/s41598-024-58941-8 (PMC11379806; doi:10.1038/s41598-024-58941-8)
Supplement: Supplementary file 1 — Supplementary Information. [file 41598_2024_58941_MOESM1_ESM.pdf]

Online supplementary materials

Using a flashlight-contingent window paradigm to investigate visual search and object memory in virtual reality and on computer screens. Beitner, Helbing, David, & Võ. (2024).

Here, we report the results of the analysis of the rebuilding performance accounting for object recognition accuracy (Table S1 & S2) and include the untruncated figure (Figure S1). Result tables are based on Hoffman and Rovine[1]. Marginal and conditional R^2 are estimated using the method implemented in the MuMIn package[2].

Supplementary figure 1

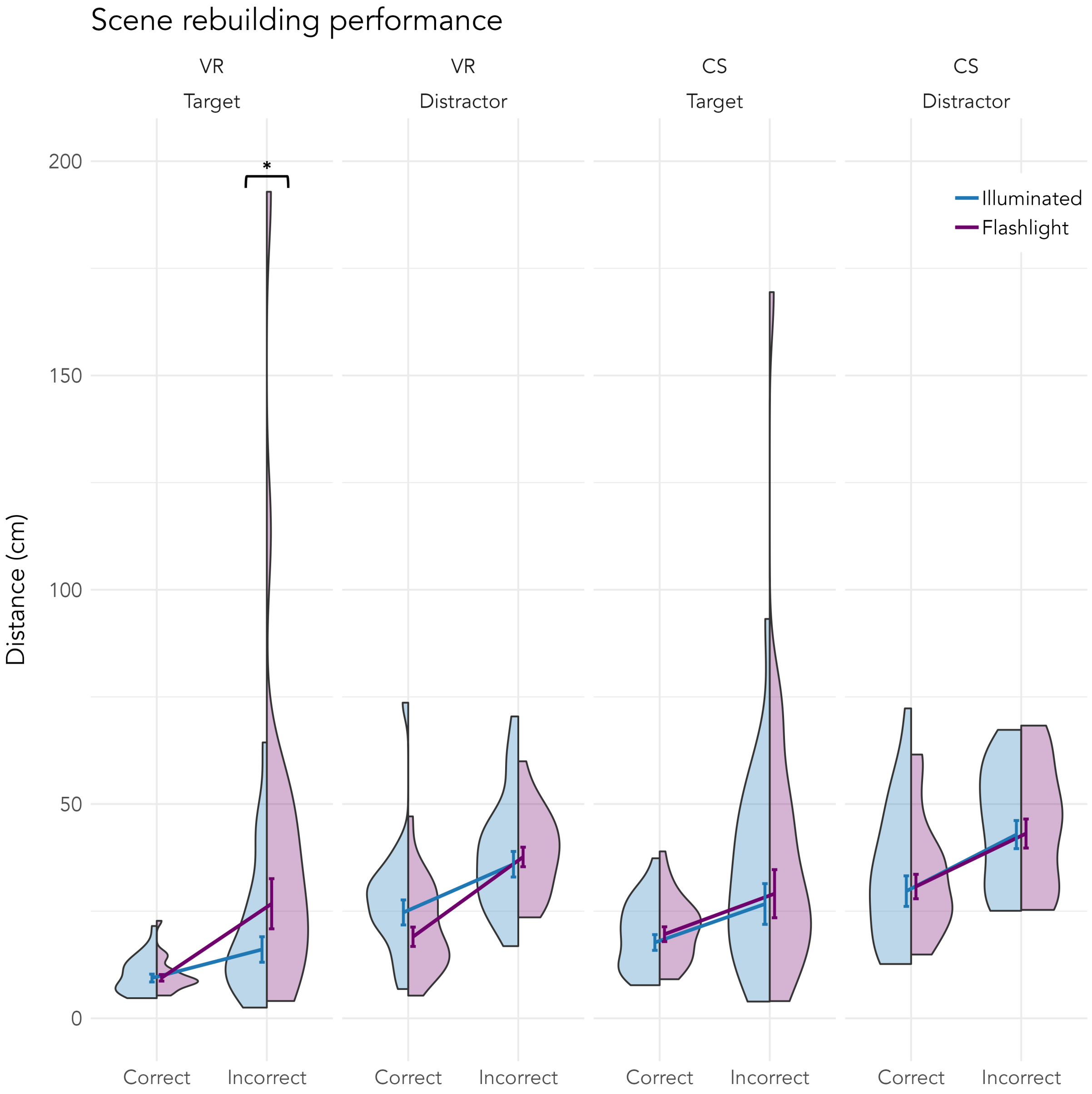

Figure S1. Incidental location memory of objects split into correct and incorrect recognition in the object recognition task. Distance from the original location in centimeters in the scene rebuilding task. Error bars indicate standard errors around the mean calculated on log-transformed distances, which were converted back to their original form for visualization purposes. CS = computer screen, VR = virtual reality. $*p < .05$

.

Supplementary analysis 1

To model scene rebuilding accuracy (measured as distance to the original location of an object), we calculated linear mixed-effects models using a Gaussian distribution and log-transformed distance measures (results in Table S1) and followed up with Bonferroni-corrected pairwise post-hoc comparisons (results in Table S2).

	VR model				CS model			
Fixed effects	Estimate	*SE*	*t*	*p*	Estimate	*SE*	*t*	*p*
Intercept	–0.92	0.07	–12.29	$< .001$	–0.54	0.08	–7.15	$< .001$
Condition (Flashlight – Illuminated)	0.06	0.09	0.59	.559	0.07	0.06	1.29	.198
Object type (Distractor – Target)	0.73	0.06	12.44	$< .001$	0.42	0.06	7.41	$< .001$
Recognition accuracy (Correct – Incorrect)	–0.59	0.06	–9.66	$< .001$	–0.42	0.06	–7.01	$< .001$
Condition × Object type	–0.33	0.12	–2.80	.005	–0.05	0.11	–0.41	.685
Condition × Recognition accuracy	–0.38	0.12	–3.24	.001	0.06	0.11	0.51	.611
Object type × Recognition accuracy	0.33	0.12	2.78	.005	0.17	0.12	1.50	.134
Condition × Object type × Recog. acc.	0.19	0.24	0.79	.432	0.14	0.23	0.61	.543
Variance components	Variance	*SD*			Variance	*SD*		
Object – Intercept	0.32	0.57			0.28	0.53		
Participant – Intercept	0.04	0.20			0.06	0.24		
– Condition	0.10	0.32			0.00	0.02		
Residual	1.57	1.25			1.18	1.09		
Pseudo-R^2($R^2_{marginal}$, $R^2_{conditional}$)	.14, .31				.08, .28			
	Full model							
Fixed effects	Estimate	*SE*	*t*	*p*				
Intercept	–0.73	0.06	–11.63	$< .001$				
Condition (Flashlight – Illuminated)	0.08	0.05	1.41	.163				
Object type (Distractor – Target)	0.58	0.04	14.12	$< .001$				
Experiment (CS – VR)	0.38	0.08	4.75	$< .001$				
Recognition accuracy (Correct – Incorrect)	–0.49	0.04	–11.36	$< .001$				
Condition × Object type	–0.19	0.08	–2.27	.024				
Condition × Experiment	0.01	0.11	0.10	.919				
Object type × Experiment	–0.30	0.08	–3.65	$< .001$				
Condition × Recognition accuracy	–0.15	0.08	–1.86	.063				
Object type × Recognition accuracy	0.27	0.08	3.23	.001				
Experiment × Recognition accuracy	0.19	0.08	2.28	.023				
Condition × Object type × Experiment	0.25	0.16	1.53	.126				
Condition × Object type × Recog. acc.	0.21	0.17	1.24	.216				
Condition × Experiment × Recog. acc.	0.43	0.17	2.58	.010				
Object type × Experiment × Recog. acc.	–0.14	0.17	–0.82	.414				
Cond. × Obj. type × Exp. × Recog. acc.	0.02	0.33	0.05	.958				
Variance components	Variance	*SD*						
Object – Intercept	0.28	0.53						
Participant – Intercept	0.05	0.22						
– Condition	0.05	0.23						
Residual	1.39	1.18						
Pseudo-R^2($R^2_{marginal}$, $R^2_{conditional}$)	.13, .30							

Table S1. Results of the linear mixed-effects model for log-transformed distances of placed objects in the scene rebuilding task including estimated regression coefficients. CS = computer screen, VR = virtual reality.

Contrast: Incorrect – Correct	VR				CS			
Condition, Object type	Estimate	*SE*	*z*	*p*	Estimate	*SE*	*z*	*p*
Illuminated, Target	0.52	0.12	4.39	< .001	0.47	0.15	3.04	.002
Illuminated, Distractor	0.28	0.11	2.62	.009	0.37	0.10	3.78	< .001
Flashlight, Target	0.98	0.12	8.33	< .001	0.52	0.14	3.81	< .001
Flashlight, Distractor	0.55	0.10	5.47	< .001	0.21	0.10	2.09	.037
Contrast: Illuminated – Flashlight	VR				CS			
Object type, Recognition accuracy	Estimate	*SE*	*z*	*p*	Estimate	*SE*	*z*	*p*
Target, Correct	0.01	0.09	0.07	.948	–0.09	0.09	–1.02	.308
Target, Incorrect	–0.46	0.15	–3.00	.003	–0.14	0.20	–0.69	.491
Distractor, Correct	0.22	0.12	1.77	.078	–0.14	0.11	–1.22	.221
Distractor, Incorrect	–0.05	0.10	–0.49	.627	0.03	0.11	0.30	.768

Table S2. Bonferroni-adjusted post-hoc comparisons of the rebuilding data accounting for recognition accuracy. CS = computer screen, VR = virtual reality.

References

1. Hoffman, L. & Rovine, M. J. Multilevel models for the experimental psychologist: Foundations and illustrative examples. *Behav. Res. Methods* **39**, 101–117, DOI: 10.3758/BF03192848 (2007).

2. Nakagawa, S. & Schielzeth, H. A general and simple method for obtaining R^2 from generalized linear mixed-effects models. *Methods Ecol. Evol.* **4**, 133–142, DOI: 10.1111/j.2041-210x.2012.00261.x (2013).
